# Evaluación morfométrica del complejo estilohioideo mediante tomografía computarizada multicorte

**DOI:** 10.21142/2523-2754-0902-2021-056

**Published:** 2021-06-21

**Authors:** Mariana Raquel Hernández-Díaz, Adalsa Hernández-Andara, Ana Isabel Ortega-Pertuz

**Affiliations:** 1 Facultad de Odontología, Universidad Central de Venezuela. Caracas, Venezuela. maryhernandez29@gmail.com Universidad Central de Venezuela Facultad de Odontología Universidad Central de Venezuela Caracas Venezuela maryhernandez29@gmail.com; 2 Unidad de Diagnóstico por Imagen, Clínica Félix Boada. Caracas, Venezuela. adasa1@yahoo.com Unidad de Diagnóstico por Imagen Clínica Félix Boada Caracas Venezuela adasa1@yahoo.com; 3 Facultad de Odontología, Universidad del Zulia. Maracaibo, Venezuela. anitaortegav@gmail.com Universidad del Zulia Facultad de Odontología Universidad del Zulia Maracaibo Venezuela anitaortegav@gmail.com

**Keywords:** hueso temporal, hueso hioides, tomografía computarizada multidetector, temporal bone, hyoid bone, multidetector computed tomography

## Abstract

**Objetivo:**

Estudiar morfométricamente el complejo estilohioideo (CEH) mediante tomografía computarizada multicorte (TCM).

**Materiales y métodos:**

Investigación descriptiva, retrospectiva y transversal. La muestra estuvo conformada por 238 estudios de TCM, pertenecientes a pacientes de ambos sexos con edades entre 20 y 87 años, con indicación de tomografía para el macizo craneofacial. Se realizó la medición de la longitud del CEH en vistas laterales de reconstrucciones volumétricas en 3D. Se obtuvo la distribución de estos casos de acuerdo con edad, sexo, lateralidad, tipo de osificación y motivo de indicación del examen.

**Resultados:**

La longitud media del CEH fue de 30,66 ± 10,58 mm. Del total de la muestra, 127 (53,4%) individuos mostraron un CEH elongado; de estos, un 63,8% fueron mujeres y un 64,6% de los pacientes presentó un compromiso bilateral del complejo. La mayoría de los sujetos con un CEH elongado tenían edades comprendidas entre 30 y 59 años. El tipo de osificación encontrada con mayor frecuencia fue del tipo I (elongación continua). En cuanto al motivo de indicación del examen, la mayoría de los pacientes fueron referidos para estudio de la articulación temporomandibular.

**Conclusiones:**

La TCM es una herramienta útil en la identificación y el estudio morfométrico de la osificación del CEH, tanto en su longitud como tipo. El examen de un CEH osificado es importante para el diagnóstico diferencial de dolor cervicofacial y disfunción de la articulación temporomandibular.

## Abstract (en)

**Objective:**

tomography (MCT).

**Materials and methods:**

This was a descriptive, retrospective cross-sectional study. The sample was made up of 238 MCT studies performed in patients of both sexes between 20 to 87 years of age, with indication of tomography of the craniofacial complex. The length of the SHC was measured in lateral views of 3D volumetric reconstructions. The distribution of these cases was obtained according to age, sex, laterality, type and the reason for the examination.

**Results:**

The mean length of the SHC was 30.66 ± 10.58 mm. Of the total sample, 127 (53.4%) individuals showed an elongated SHC; of these, 63.8% were women and 64.6% of the patients presented a bilateral compromise of the complex. Most of the subjects with an elongated SHC were between 30 and 59 years old. The type of ossification most frequently found was type I (continuous elongation). Regarding the reason for the examination, most of the individuals were referred for study of the temporomandibular joint.

**Conclusions:**

MCT is a useful tool for the identification and morphometric study of ossification of the SHC, both in length and type. Examination of an ossified SHC is important for the differential diagnosis of cervico-facial pain and temporomandibular joint dysfunction.

## INTRODUCCIÓN

La apófisis estiloides (AE), el ligamento estilohioideo (LEH) y el cuerno menor del hueso hioides (HH) forman un complejo anatómico multiestructural llamado complejo estilohioideo (CEH). La AE constituye una proyección ósea delgada, larga y cilíndrica ^1^^-^^3^ que emerge proximalmente desde la porción timpánica del temporal, mientras que su extremo distal proporciona inserción a los músculos estilofaringeo, estilohioideo y estilogloso, así como a los ligamentos LEH y estilomandibular; estas estructuras regulan el movimiento de la lengua, la faringe y el HH ^4^^,^^5^. La apófisis se proyecta anterior, inferior y medialmente se localiza entre las arterias carótidas interna/externa y la vena yugular interna, y anteriormente al foramen estilomastoideo ^1^^-^^3^. El LEH es una banda de tejido conectivo que se origina en el vértice de la AE y se inserta en el cuerno menor del HH ^2^.

El CEH deriva del cartílago de Reichert y consiste en cuatro partes: timpanohial, estilohial, ceratohial e hipohial ^3^^,^^4^^,^^6^^,^^7^. La porción timpanohial, presente en el nacimiento, desarrolla la base de la AE y se calcifica en el primer año de vida; la porción estilohial se transforma en el segmento principal de la apófisis, aparece después del nacimiento y se fusiona con la porción timpanohial en la pubertad ^4^^,^^6^; por su parte, la porción ceratohial forma el LEH. Por último, la porción hipohial madura en el cuerno menor del HH ^6^.

Las variaciones anatómicas del CEH incluyen alteraciones en la longitud de la AE (ausente, duplicada o alongada), varios grados de osificación del LEH, así como la fusión de sus porciones osificadas ^8^^,^^9^. Se considera que una AE normal mide 25 mm, de manera que su elongación puede ser asumida si la apófisis o el LEH osificado muestran una longitud mayor a 30 mm ^1^^,^^4^^,^^7^^,^^8^^,^^10^^-^^12^. Sin embargo, la longitud de la apófisis es muy variable: de 5-50 mm ^1^^,^^10^^,^^11^. La prevalencia de una AE alargada está entre el 2% y el 30%, con una leve predilección por el sexo masculino ^1^^,^^3^^,^^9^^,^^12^^,^^13^, generalmente de aparición bilateral ^1^^,^^3^^,^^5^^,^^14^^,^^15^, y es detectada como un hallazgo incidental en radiografías alrededor de los 40 años ^9^^,^^16^. Clínicamente, una AE alongada puede palparse en la región de la fosa tonsilar ^12^; los pacientes son, por lo general, asintomáticos y solo un 4% de ellos refieren síntomas ^2^.

El LEH puede osificarse debido a una irritación local crónica, trauma quirúrgico, desórdenes endocrinos asociados con la menopausia, persistencia de elementos mesenquimales, estrés mecánico o trauma durante el desarrollo de la AE, lo que puede resultar en una hiperplasia calcificada de la apófisis ^1^^,^^2^. Existen varias teorías que explican la osificación del CEH, entre ellas la metaplasia o hiperplasia reactiva, anomalía desarrollada con la edad, y la metaplasia intraligamentaria ^1^^,^^5^^,^^8^. La teoría congénita, la cual es la más aceptada, indica que el estrés mecánico puede algunas veces conducir al estiramiento del cartílago de Reichert y la osificación del LEH durante el desarrollo de la AE ^5^. 

Debido a la posición estratégica del CEH, cualquier anomalía en su estructura puede conducir a síntomas inespecíficos, que incluyen dolor faríngeo en la fosa tonsilar, otalgia, sensación de cuerpo extraño y cambios en la voz, los cuales, considerados en conjunto, constituyen el síndrome de Eagle. Estos síntomas pueden ser confundidos con otras enfermedades como neuralgias faciales, temporomandibulares, bucales o dentales ^3^^,^^8^^,^^13^. Por ello, la exploración clínica de los pacientes con dolor cervicofacial y disfunción temporomandibular debe incluir la investigación del CEH, de manera que el conocimiento detallado de sus variaciones anatómicas y posibles efectos en las estructuras suprahioideas es importante para un diagnóstico preciso ^3^.

La osificación del CEH ha sido evaluada por medio de estudios 2D como ortopantomografías dentales, cefálicas laterales, laterales de mandíbula o anteroposteriores de cráneo, así como por métodos tomográficos o anatómicos en cráneos secos ^2^^,^^3^^,^^5^^,^^6^^,^^11^^,^^15^^,^^17^^-^^21^, mediante diversos protocolos de medición de acuerdo con la técnica imagenológica empleada, lo que puede conducir a resultados variables. 

De acuerdo con lo expuesto, el propósito de este trabajo fue estudiar morfométricamente el CEH mediante tomografía computarizada multicorte (TCM), con énfasis en la presencia de osificación, longitud y tipo, para establecer un protocolo de segmentación y reconstrucción volumétrica de la AE.

## MATERIALES Y MÉTODOS

Esta investigación fue descriptiva, retrospectiva, de campo y transversal. La muestra estuvo constituida por estudios tomográficos pertenecientes a pacientes de ambos sexos, mayores de 20 años, que se realizaron una TCM del macizo craneofacial, asintomáticos o no, en una clínica privada, durante el periodo de un año. Se obtuvo de cada individuo datos demográficos (edad, sexo), motivo de indicación del examen y se estudiaron, en ambos CEH, su longitud, lateralidad y tipo de osificación. Adicionalmente, se indagó sobre el motivo de indicación del examen tomográfico, para conocer si un CEH osificado o un diagnóstico presuntivo de síndrome de Eagle fue considerado entre las posibles causas de la sintomatología dolorosa referida por el paciente. 

Para el empleo de las imágenes en este trabajo, se obtuvo el consentimiento informado de cada paciente. El estudio tomográfico fue realizado con fines de diagnóstico y orientado desde los hallazgos clínicos, y no para uso exclusivo de la presente investigación, de acuerdo con la declaración de Helsinki ^22^ sobre investigación en humanos. Asimismo, se garantizó el anonimato de cada sujeto. 

### Obtención de las imágenes mediante TCM

Las imágenes fueron obtenidas utilizando un equipo tomográfico multicorte de 16 canales (Brightspeed, GE Healthcare, WI, EE. UU.), con el paciente en posición supina, y se empleó un soporte para la ubicación de cráneo, orientado por el plano de Camper, perpendicular a la mesa del tomógrafo. No fue necesaria la preparación previa del individuo. Las imágenes fueron adquiridas utilizando los siguientes parámetros: espesor de corte de 0,625 mm, Pitch de 0,3 mm, 120 Kv, 100 mA, FOV para cabeza, con filtro de tejido óseo y formato DICOM (*Digital Imaging and Communications in Medicine*). Las imágenes fueron analizadas por un mismo examinador, un radiólogo maxilofacial con más de 15 años de experiencia en el área, que empleó una estación de trabajo Advantage (AW Volume, Share 5, GE Healthcare, WI, EE. UU.) para obtener imágenes tanto en MPR (*Multiplanar Reconstruction*) como en 3D.

### Evaluación de las imágenes

#### Medición del CEH

**Figura 1:**
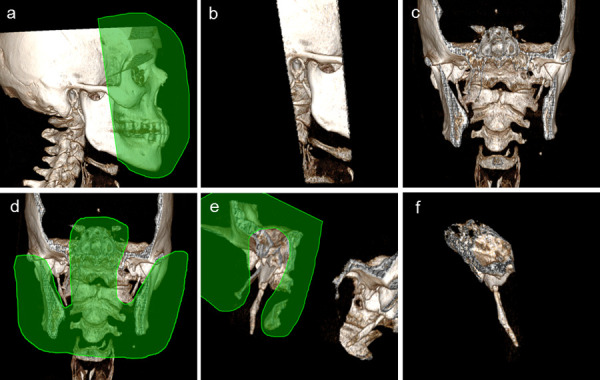
Reconstrucciones en 3D de TCM que muestran el procedimiento de obtención de la imagen coronal, para lo cual se realizaron las mediciones del complejo estilohioideo. Se muestra lo siguiente: a) Vista lateral del cráneo; b) Eliminación de las estructuras anteriores y posteriores; c) Vista coronal de la imagen obtenida; d) y e) Eliminación de las estructuras laterales y mediales de la apófisis estiloides; f) Vista coronal de la apófisis después del proceso de segmentación de la imagen original.

**Figura 2:**
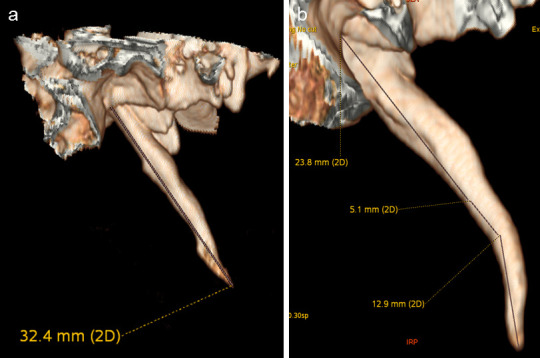
Vista lateral de reconstrucciones en 3D de TCM, donde se muestra lo siguiente: a) Medición de la apófisis estiloides, desde su base en el hueso temporal hasta el extremo distal; b) Procedimiento cuando la apófisis mostraba una flexión o curvatura.

Para la obtención de la longitud del CEH, se consideraron las reconstrucciones en 3D. En una vista lateral, se suprimieron las estructuras anatómicas anteriores, posteriores, laterales y mediales a la AE, hasta obtener una vista completa (Figura 1). La medición se realizó desde la base de la apófisis hasta la última porción detectable en la tomografía (Figura 2). En los casos en los que la osificación del CEH se encontraba segmentada, las partes no osificadas fueron medidas e incluidas en dicha longitud; asimismo, en aquellos CEH con flexiones o curvaturas, cada porción fue medida y su longitud sumada para obtener un valor total del CEH (Figura 2). Las medidas se realizaron de forma bilateral. La AE fue considerada alongada cuando media más de 30 mm, en concordancia con lo descrito por la literatura ^1^^,^^4^^,^^8^^,^^10^^,^^15^.

#### Valoración de la morfología

La morfología de la osificación del CEH fue evaluada mediante la clasificación de Langlais *et al.*^23^, como sigue:

• Tipo I o alongado: este patrón representa un AE alongada ininterrumpida. La AE y el LEH aparecen como una estructura continua. 

• Tipo II o pseudoarticulado: se caracteriza por una AE aparentemente unida al LEH mediante una pseudoarticulación única, que está usualmente localizada en la parte superior, a un nivel tangencial del borde inferior de la mandíbula. Esto da una visión general de un CEH alongado aparentemente articulado. 

• Tipo III o segmentado: constituido por segmentos cortos o largos interrumpidos de ligamento mineralizado, lo que crea la apariencia de pseudo-articulaciones múltiples en el ligamento. En cualquiera de los casos, se observan dos o más segmentos, con interrupciones por encima o por debajo del borde inferior de la mandíbula, o en ambos. 

#### Análisis estadístico

Para el análisis de los datos, se empleó el *software* IBM SPSS Statistic, versión 23 (IBM Corp, Armonk, NY, EE. UU.) para Windows. Se calcularon estadísticos descriptivos (medias, desviación estándar) de la edad en la cual se observó la osificación del CEH, así como frecuencias y porcentajes de la distribución por grupo de edad, sexo, lateralidad, tipo de osificación y motivo de consulta del examen. 

## RESULTADOS

Se obtuvo una muestra de 238 estudios tomográficos, de los cuales 155 (65,1%) pertenecían al sexo femenino y 83 (34,9%) al masculino. El rango de edad de los pacientes evaluados fue de los 20 a los 87 años, con una media de 44,22 ± 16,10 años. Por otra parte, se observó que el 75% de los pacientes presentan una edad inferior a 56 años. Del total de la muestra estudiada, el 53,4% (n = 127) de los individuos presentó una AE alongada, mientras que el 46,6% (n = 111) tenía una apófisis con una longitud inferior a 30 mm. Con respecto al sexo, la AE alongada se observó con un mayor porcentaje en el sexo femenino (63,8%, n = 81) que en el masculino (36,2%, n = 46). En cuanto a la lateralidad, se verificó que, de los 127 afectados, 24 (18,9%) presentaron una AE alongada en el lado derecho; 21 (16,5%), en el lado izquierdo, y en 82 (64,6%) se observó de forma bilateral. 

La longitud del CEH, independientemente de la lateralidad, tuvo un valor medio de 30,64 ± 10,58 mm, y varió entre 3,9 y 86,4 mm. La longitud del CEH tuvo una media de 30,66 ± 10,49 mm en el lado derecho (3,9 a 86,40 mm) y, para el lado izquierdo, de 30,62 ± 10,69 mm (6,7 a 85,5 mm). Dentro del conjunto de los individuos que mostraron una AE alongada y/u osificación del LEH, se obtuvo una longitud media de 38,83 ± 9,75 mm, que varió entre 30,1 y 86,4 mm. Para el lado derecho, este valor fue de 38,71 ± 9,46 mm, con un rango entre 30,1 y 86,4 mm, y para el lado izquierdo, de 38,95 ± 10,08 mm, con una variación de 30,1 a 85,5 mm.

Con relación a la distribución de la AE alongada de acuerdo con el grupo de edad e independiente del sexo (Tabla 1), se verificó que la mayoría de los pacientes afectados se encontraba entre la tercera y quinta década de vida. La Tabla 2 muestra los resultados obtenidos para el tipo de osificación y evidencia que el tipo I fue el más frecuente (57,6%, n = 137), seguido por el tipo III (36,1%, n = 86) y el tipo II (6,3%, n = 15). Al relacionar el tipo de osificación según el lado, se encontró que la aparición bilateral del tipo I fue la más frecuente, seguida por la tipo III y la combinación tipo I derecho con tipo III izquierdo. 

**Tabla 1:** Distribución del complejo estolohioideo osificado según los grupos de edad

Grupo de edad		Complejo estolohioideo osificado		Total
		Sí (n)	No (n)	
≤29		22	34	56
	Dentro de grupo de edad (%)	39,3	60,7	100,0
	En la muestra total (%)	17,3	30,6	23,5
30-39		32	19	51
	Dentro de grupo de edad (%)	62,7	37,3	100,0
	En la muestra total (%)	25,2	17,1	21,4
40-49		23	16	39
	Dentro de grupo de edad (%)	59,0	41,0	100,0
	En la muestra total (%)	18,1	14,4	16,4
50-59		29	17	46
	Dentro de grupo de edad (%)	63,0	37,0	100,0
	En la muestra total (%)	22,8	15,3	19,3
60-69		13	16	29
	Dentro de grupo de edad (%)	44,8	55,2	100,0
	En la muestra total (%)	10,2	14,4	12,2
≥70		8	9	17
	Dentro de grupo de edad (%)	47,1	52,9	100,0
	En la muestra total (%)	6,3	8,1	7,1
Total		127	111	238
	Dentro de grupo de edad (%)	53,4	46,6	100,0
	En la muestra total (%)	100,0	100,0	100,0

**Tabla 2:** Distribución del tipo de osificación del complejo estilohioideo de acuerdo con la lateralidad

Lado				Total
Derecho	Izquierdo				
	Tipo I	Tipo II	Tipo III		
Tipo I	120	5	12	137
	50,4%	2,1%	5,0%	57,6%
Tipo II	6	5	5	16
	2,5%	2,1%	2,1	6,7
Tipo III	115	5	69	85
	4,6%	2,1%	29,0%	35,7%
Total	137	15	86	238
	57,6%	6,3	36,1	100,0

Tipo I: elongado; Tipo II: pseudoarticulado; Tipo III: segmentado

La Tabla 3 presenta la distribución de la muestra de acuerdo con el motivo de indicación del examen y sexo. En la mayoría de los casos, el examen fue solicitado por sospecha de disfunción de la ATM, seguido por la evaluación de patologías, la planificación de implantes y la valoración de trauma facial. Cuando esto fue considerado por sexo, se observó en las mujeres una tendencia similar a la verificada para el total de la muestra, mientras que, en los hombres, la mayor frecuencia se presentó con relación a la evaluación de patologías, la sospecha de disfunción de la ATM y el trauma facial.

**Tabla 3:** Distribución de la muestra de acuerdo con la indicación del examen y el sexo

Motivo de indicación del examen	Sexo						
	Femenino			Masculino		Total	
	n	%	n	%	n	%
Articulación temporomandibular	103	43,3	24	10,1	127	53,4
Cirugía ortognática	2	0,8	0	0,0	2	0,8
Hendidura palatina	0	0,0	1	0,4	1	0,4
Planificación de implante	19	8,0	8	3,4	27	11,3
Localización de estructuras dentarias	6	2,5	6	2,5	12	5,0
Patología	21	8,8	25	10,5	46	19,3
Trauma facial	4	1,7	19	8,0	23	9,7
Total	155	65,1	83	34,9	238	100,0

## DISCUSIÓN

El CEH es abundante en variaciones anatómicas, esto incluye su longitud y diversos grados de osificación ^8^^,^^9^^,^^15^^,^^24^. La AE se osifica de cinco a ocho años después del nacimiento ^6^ y a la edad de 20 años su longitud se estabiliza ^3^ Las apófisis estiloides más largas resultan de la osificación de las porciones timpanohial y estilohial durante los primeros años de vida, mientras que una AE más corta se produce por la osificación única de la porción timpanohial ^24^. Normalmente, las porciones ceratohial e hipohial no se osifican y, si lo están, se fusionan durante la pubertad tardía o el inicio de la adolescencia; por tanto, distintos tipos de CEH osificados pueden estar presentes durante la vida y conducir a una marcada variación en la apariencia radiográfica del complejo ^4^^,^^6^. 

Varias estructuras neurovasculares se encuentran próximas a la punta de la AE, entre ellas la arteria carótida interna/externa, la vena yugular interna y los nervios craneales X, XI y XII, en su lado medial ^1^^,^^2^. Se ha reportado que los mecanismos fisiopatológicos del dolor asociado con un CEH osificado, conocido como síndrome de Eagle, están relacionados con lo siguiente: a) Compresión de elementos neurales; b) Fractura del LEH seguida por una reacción inflamatoria; c) Punción de los vasos carotideos que produce irritación de los nervios simpáticos en la vaina arterial; d) Cambios degenerativos o inflamatorios en la porción tendinosa de la inserción del LEH; e) Irritación de la mucosa faríngea por compresión directa; y f) Estiramiento y fibrosis de los pares craneales IV, VII y IX, después de una tonsilectomía ^18^^,^^19^.

La prevalencia observada de la osificación del CEH fue del 53,40%, valor fue superior al reportado por Yilmaz *et al.*^25^ (30%), quienes emplearon la tomografía computarizada para angiografía carotidea, e inferior a lo verificado por Buyuk *et al.*^5^ (63,95%), en imágenes de tomografía computarizada de haz cónico (TCHC), y Ekisi *et al.*^15^ (56%), en TCM. Cuando se consideraron estudios realizados en ortopantomografías dentales, esta prevalencia fue superior a la verificada por Alpoz *et al.*^2^ (27,1%) y Krennmair *et al.*^7^ (30,7%); inferior a la observada por Ledesma-Montes *et al.*^26^ (43,60%) y Vieira *et al.*^16^ (43,89%); y similar a la verificada por Zokaris *et al.*^3^ (30%). 

La longitud media encontrada de la AE en este estudio, considerando la apófisis por sí sola o en combinación con un LEH osificado, fue de 30,64 mm para el total de la muestra y en ambos lados. En trabajos en los que se emplearon métodos tomográficos (TCHC o TCM), se han reportado longitudes medias superiores, como en las investigaciones de Bujuk *et al.* ^(5^) (45,89 mm) y Andrade *et al.*^14^ (35,09 mm), mientras que la media observada por Ekici *et al.*^15^ fue similar (31,2 mm). Sin embargo, dicha longitud media fue superior a la observada por Başekin *et al.*^6^ (28,4 mm), Cullu *et al.*^27^ (28,4 mm), Gözil *et al.*^12^ (27,42 mm), Ilgüy *et al.*^28^ (25,3 mm), Onbas *et al.*^17^ (26,8 mm) y Shayganfar *et al.*^21^ (25,3 mm). 

Los distintos valores de longitud y prevalencia encontrados pueden ser atribuidos no solo a características poblacionales ^21^^,^^27^, sino también a los distintos métodos empleados para realizar las mediciones del CEH osificado, lo que es crítico en las ortopantomografías, en cuyo caso los factores inherentes a la geometría de proyección, la distorsión y la magnificación de imagen también deben ser considerados como elementos capaces de disminuir la precisión en la identificación de un CEH osificado ^1^^,^^13^^,^^15^^,^^19^^,^^29^. Otros factores por considerar son el valor asumido para una AE normal ^6^, así como discrepancias en la estructura (grupos de edad y sexo) y el tamaño de la muestra ^21^.

En relación al sexo, las mujeres tuvieron una mayor frecuencia de casos de osificación del CEH (63,8%), lo que concuerda con los estudios de Chabikuli *et al.*^4^, Ilgüy *et al.*^28^, Ledesma-Montes *et al.*^26^, Okur *et al.*^18^ y Viera *et al.*^16^, pero se contrapone a lo encontrado por AlZarea *et al.*^11^, Bagga *et al.*^1^, Gözil *et al.*^12^, Kailasam *et al.*^13^, Katti *et al.*^9^ y Zokaris *et al.*^3^. Esto puede estar relacionado con que la mayoría de los pacientes de la muestra fueron remitidos por sospecha de disfunción de ATM y esta tiene una frecuencia alta en el sexo femenino ^30^. Con respecto a la edad, se evidenció que los casos de CEH osificado se observaron en mayor número entre la tercera y quinta década de vida. La media de la edad de los pacientes de dichos casos fue alrededor de los 40 años, lo que coincide con estudios previos ^9^^,^^13^^,^^16^^,^^18^. 

En concordancia con otras investigaciones ^1^^,^^3^^,^^5^^,^^13^^,^^15^, la mayoría de los casos de osificación del CEH hallados en el presente trabajo fueron bilaterales, lo cual puede ser atribuido al hecho de que una actividad extenuante y hábitos masticatorios conducen a la contractura de los músculos de la masticación; esto aumenta la carga para la AE y promueve la osificación ^1^. En cuanto al tipo de osificación, el tipo I fue el más frecuente, lo que coincide con los trabajos de AlZarea *et al.*^11^, Buyuk *et al.*^5^, Chabikuli *et al.*^4^, de Andrade *et al.*^14^, Kashyap *et al.*^8^, Katti *et al.*^13^ y Öztunç *et al.*^19^.

Los estudios tomográficos proporcionan mediciones precisas y permiten analizar la geometría espacial de las estructuras anatómicas ^21^^,^^27^^,^^30^; en ese sentido, la reconstrucción volumétrica en 3D es el estándar de oro para la evaluación morfométrica del CEH ^15^^,^^27^. Al analizar los protocolos empleados en las diferentes investigaciones que incluyen métodos tomográficos para el estudio del CEH ^5^^,^^6^^,^^15^^,^^17^^-^^25^, pueden hacerse varias generalizaciones: a) Las mediciones son realizadas desde la base de la AE en el hueso temporal hasta el extremo de la última porción osificada; b) Dichas mediciones incluyen los segmentos no osificados como parte de la longitud total del complejo; c) Se emplearon reconstrucciones volumétricas en 3D, en vistas laterales o coronales; y d) Las reconstrucciones multiplanares constituyen un auxilio para la localización de las referencias anatómicas necesarias para la medición. Creemos que el protocolo de segmentación presentado en este trabajo facilita la localización de las referencias anatómicas para la medición del CEH, particularmente, la vista lateral proporcionó una observación completa de la estructura. 

Entre los posibles diagnósticos diferenciales de la osificación del CEH sintomática (síndrome de Eagle) se encuentran la disfunción temporomandibular, neuralgia del trigémino, nervio esfenopalatino o glosofaríngeo, migrañas, arteritis de la temporal, bursitis del HH, infecciones crónicas (faringotonsilitis), otitis media, dolor de origen dental, una prótesis mal adaptada, desórdenes de glándulas salivares o sialolitiasis, presencia de cuerpos extraños o tumores, disestesia laringofaríngea, síndrome de Sluder o diverticulitis esofágica ^20^^,^^29^. 

Se cree que entre el 4% y el 75% de la población tiene al menos un signo de disfunción temporomandibular y el 33%, al menos un síntoma. Este término engloba un gran número de signos y síntomas similares a otras condiciones, entre ellos el dolor preauricular, los ruidos en la articulación, el dolor referido, la cefalea, el tinnitus, la otalgia, el vértigo, la sensación de obstrucción en el oído y la hipo o hiperacusia, los cuales pueden ser semejantes a los referidos por pacientes con un CEH osificado sintomático ^14^. No se ha encontrado asociación entre la disfunción temporomandibular y una AE alongada, es más probable que estas condiciones coexistan debido al disturbio muscular asociado con ambas ^24^. La frecuencia de la disfunción podría explicar el mayor número de pacientes referidos para examen de la ATM en este estudio, con respecto a otras indicaciones. Es importante puntualizar que ninguno de los pacientes fue remitido para evaluación mediante TCM por sospecha de síndrome de Eagle. 

El tratamiento de la osificación del CEH puede ser convencional (analgésicos y corticoesteroides) o quirúrgico; este último puede realizarse mediante un abordaje extra o intrabucal ^20^. El abordaje extrabucal proporciona mayor visibilidad y menor riesgo de infección bacteriana; sin embargo, su principal desventaja es que requiere anestesia general y la cicatriz puede constituir un problema estético. El abordaje intrabucal es más simple que el extrabucal, requiere un menor tiempo quirúrgico y evita cicatrices externas, pero existe mayor riesgo de injuria neurovascular e infecciones profundas en el cuello ^15^^,^^20^.

## CONCLUSIÓN

La TCM, es una herramienta útil para la identificación y el estudio morfométrico de la osificación del CEH, tanto en su longitud como en su tipo, ya que la vista lateral segmentada de la reconstrucción volumétrica en 3D de la AE y/o un ligamento estilohioideo osificado ofrece una completa visualización del complejo. Dicha osificación tuvo una alta frecuencia en la muestra estudiada, alrededor de los 40 años de edad y de tipo I (elongada). El examen de un CEH osificado es importante para el diagnóstico diferencial de dolor cervicofacial y la disfunción de la articulación temporomandibular.

## Fuente de financiamiento:

Autofinanciado
